# 2367: Defining critical features of the immune microenvironment in melanoma using multiplex immunohistochemistry and spatial analysis

**DOI:** 10.1017/cts.2017.224

**Published:** 2018-05-10

**Authors:** Robyn Gartrell, Douglas Marks, Thomas Hart, Yan Lu, Ed Stack, Camden Esancy, Basil Horst, Yvonne Saenger, Camille Gerard, Dan Tong Jia, Paul Armenta, Daisuke Izaki, Kristen Beck

**Affiliations:** Irving Institute for Clinical, Columbia University, New York, NY, USA

## Abstract

OBJECTIVES/SPECIFIC AIMS: Precise biomarkers are urgently needed to characterize the tumor immune microenvironment in primary melanoma tumors both for prognostication and to predict the benefit of immuno-therapeutic intervention. The goal of this work is to define spatial relationships between CD8+ T cells, CD68+ macrophages and Sox10+ melanoma cells in order to define features correlating with prolonged survival METHODS/STUDY POPULATION: Five micrometer slides from either the primary biopsy or subsequent wide local excision procedure were stained using Opal multiplex IHC for DAPI, CD3 (LN10, Leica), CD8 (4B11, Leica), CD68 (KP1, Biogenex), SOX10 (BC34, Biocare), HLA-DR (LN-3, Abcam), and Ki67 (MIB1, Abcam). Cell phenotypes within representative fields preselected by a trained dermato-pathologist and were visualized using the Mantra quantitative pathology workstation (PerkinElmer), and analysis of spatial distribution of CD3+ CD8+ cells analyzed using inForm^®^ image analysis software (PerkinElmer), and Spotfire software (TIBCO). In order to test whether mIHC can better characterize the tumor immune microenvironment, we screened databases at the Herbert Irving Cancer Center (HICC) at Columbia University for stage II/III melanoma patients diagnosed between 2000 and 2012, with available FFPE of primary melanoma tissue and documented clinical follow-up. We identified a preliminary population of 57 patients to begin our analysis. Clinical follow-up was available on 35 patients of whom 21 patients were alive with no evidence of recurrence or died with no evidence of recurrence and 14 had died of melanoma. Twenty-four patients had more than 24 months of survival information available but no detailed clinical information to determine cause of death. RESULTS/ANTICIPATED RESULTS: First, we evaluated whether density of immune cells in tumor and stroma predicted prognosis in 35 patients with disease specific survival information. We find that high number of CD3+CD8+ cells in tumor correlates with Disease Specific Survival (DSS) (*p*=0.0323*) and CD3+CD8+ cells in stroma may also correlate with DSS (*p*=0.0671). This is consistent with what is known in the literature regarding tumor infiltrating lymphocytes (TILs). We also found that CD68+ cells in stroma predict poor prognosis (0.0259*). This is consist with the proposed deleterious role for macrophages in tumor progression. Next, using nearest neighbor analysis we examined the effect of HLA-DR and Ki67 expression on spatial distribution of CD3+CD8+ T cells. We find that CD8+ T cells are closer to myeloid (CD68+) cells expressing HLA-DR. This is consistent with the potential of HLA-DR expressing cells to present antigens to T cells, and suggests that T cells may preferentially interact with HLA-DR expressing myeloid cells. Conversely, we find that Ki67 expression on tumor (SOX10+) cells correlates with increased distance from CD3+CD8+ T cells relative to SOX10+Ki67-tumor cells. This finding is consistent with the observation that more advanced tumors with higher mitotic rates have decreased T cell infiltrates, and suggests that dividing melanoma cells are less likely to interact with T cells. In addition, we performed analysis to determine whether spatial relationships defined above impact prognosis. Clinical oncology follow-up was available on 35 of the 57 patients evaluated above. We compared proximity of CD3+CD8+ cells to both myeloid (CD68+) and tumor (SOX10+) cells in patients who recurred and those with no evidence of recurrence. We found that CD3+CD8+ cells in patients who had recurrence were closer to CD68+ HLA-DR− cells than in patients who had no recurrence (*t*-test, *p*=0.0377), this correlated with DSS (*p*=0.003). Conversely, distance from CD3+CD8+ to CD68+ HLA-DR+ in relationship to recurrence was not significant with a trend towards CD3+CD8+ T cells being closer in nonrecurrent patients (*t*-test, *p*=0.1362). DISCUSSION/SIGNIFICANCE OF IMPACT: Consistent with the literature, we find that densities of CD8+ T cells correlates with favorable outcomes in early stage melanoma. We also find that density of CD68+ macrophages in stroma correlates with poor outcome. If proximity is a surrogate for interaction, these data indicate that dividing, Ki67+, melanoma cells interact less with CD8+ T cells than do Ki67+ melanoma cells. Further, HLA-DR expression on CD68+ infiltrating cells likely enhances their interaction with T cells. Interestingly, on further analysis, CD3+CD8+ cells were significantly closer to CD68+ HLA-DR− cells in patients who recurred, implying that interactions between these cell types may not be favorable. This analysis demonstrates that spatial analysis may be useful in predicting prognosis in early stage melanoma, and this is the first report of this type of analysis predicting outcomes in primary tumor specimens to our knowledge. Further staining and analysis of the complete patient cohort (n=120) is ongoing.

